# Tibiofemoral rotation is related to differences in the lateral femoral condyle configuration in both normal subjects and women with hip dysplasia: a three-dimensional analysis

**DOI:** 10.1186/s12891-019-2737-3

**Published:** 2019-07-31

**Authors:** Norio Imai, Dai Miyasaka, Yuki Hirano, Hayato Suzuki, Kazuki Tsuchiya, Naoto Endo

**Affiliations:** 1 Division of Comprehensive Geriatrics in Community, Niigata University Graduate School of Medical and Dental Sciences, Niigata 9518510, 1-757, Asahimachi-dori, Niigata, Japan; 2 Department of Orthopedic Surgery, Niigata University Medical and Dental Hospital, Niigata, 9518510 Japan; 3 Division of Advanced Materials Science and Technology, Niigata University Graduate School of Science and Technology, Niigata, 9502181 Japan

**Keywords:** Tibiofemoral rotation, Femoral neck anteversion, Clinical epicondylar axis, Developmental dysplasia of the hip, Lower extremity alignment

## Abstract

**Background:**

We assessed the morphological differences in the knee joint related to knee rotation angle in patients with hip dysplasia. These results may explain the anatomy of the knee in patients with hip dysplasia and aid in planning knee surgery.

**Methods:**

We enrolled 73 women (146 legs, 35.6 ± 9.0 years) with bilateral hip dysplasia and 45 healthy women (90 legs, 49.0 ± 18.9 years) without lumbago, knee symptoms, or osteoarthritic findings of the knee or spine on plain radiographs. We examined the parameters affecting knee rotation angle, such as the condylar twist angle and femoral condyle measurements with a three-dimensional bone model using the correlation coefficients of each parameter.

**Results:**

The condylar twist angle and the length of the posterior part of the lateral femoral condyle were statistically positively correlated with knee rotation angle in both the normal (condylar twist angle: *r* = 0.286, *p* = 0.007, posterolateral: *r* = 0.429, *p* < 0.001) and developmental dysplasia of the hip groups (condylar twist angle: *r* = 0.230, *p* = 0.033, posterolateral: *r* = 0.272, *p* = 0.005). Knee rotation angle had no statistical correlation with femoral neck anteversion in the developmental dysplasia of the hip group (*r* = 0.094, *p* = 0.264), but had a statistical correlation with femoral neck anteversion in the normal group (*r* = 0.243, *p* = 0.039).

**Conclusions:**

Knee joint morphology is affected by hip dysplasia. We found that the length of the posterior part of the lateral femoral condyle was significantly positively correlated with knee rotation angle in both the normal and developmental dysplasia of the hip groups, and this finding indicates that a greater posterolateral dimension was associated with a greater knee rotation angle. These morphological knee joint differences in patients with hip dysplasia may help determine the alignment of prostheses in total knee arthroplasty.

## Background

Developmental dysplasia of the hip (DDH) is considered the most common etiology of secondary osteoarthritis of the hip [1], and notably, the incidence of DDH is higher in Asians [2]. Several investigations described the morphological features with regard to the acetabulum and the proximal femur in patients with DDH to investigate the etiology and/or prevention of osteoarthritis of the hip [3–5]. However, morphological deformities in DDH not only appear in the hip joint but also in the knee joint, such as “coxitis knee” [6, 7]. However, few reports have investigated the knee joints and their morphology in DDH patients.

Previously, two-dimensional (2D) plain X-ray was commonly used to evaluate the alignment of the lower extremity [8]. However, the position of the subject’s pelvis and lower extremities affect the measurement values with this 2D method, and this may produce measurement errors leading to reduced accuracy and duplicability [9]. Furthermore, the 2D method is unable to evaluate malalignment spatially and geometrically between the femur and tibia, such as tibiofemoral rotation, which is considered to cause some lower extremity pathologies [10, 11].

Moreover, the knee rotation angle (KRA) has previously been shown to be significantly larger in patients with DDH than in normal healthy subjects, and there was no significant correlation between femoral neck anteversion and KRA when using the three-dimensional (3D) method [7]. Li et al. [6] described that the width of the femoral condyles was smaller and observed greater asymmetry at the medial and lateral condyles in DDH patients than in normal hips. From the results obtained in these investigations, we speculated that there are some morphological differences in the knee joint related to differences in KRA, especially in the medial and/or lateral femoral condyle.

This study aimed to assess the morphological characteristics of knee joints in DDH patients and to test the hypothesis that there are several morphological abnormalities in the knee joint related to KRA. The results may explain the anatomical condition of the knee in DDH patients and become an anatomical reference for patients undergoing any knee surgery.

## Methods

### Subjects

We enrolled 118 Japanese women. The DDH group included 73 patients (146 legs) with bilateral DDH (mean age, 35.6 ± 9.0 years; body height, 158.4 ± 6.4 cm, body weight, 55.1 ± 7.4 kg, body mass index (BMI), 22.0 ± 3.1 kg/m^2^) from our institution who received curved periacetabular osteotomy [12] for treatment of early-stage osteoarthritis with DDH and whose center-edge angle was < 25° as evaluated on anteroposterior plain X-ray of the hip [13]. We excluded patients with DDH who underwent any hip surgery previously and those with stage 2–4 subluxation according to Crowe’s classification or grade 2–3 arthritic changes according to the Tönnis classification as measured on plain radiographs of the bilateral hips. We also recruited 45 women (90 legs; mean age, 49.0 ± 18.9 years; body height, 154.4 ± 6.3 cm, body weight 52.8 ± 7.7 kg, BMI, 22.2 ± 2.9 kg/m^2^) without lumbago, knee symptoms, or any osteoarthritic findings of the knee or spine on plain radiographs. These healthy, normal subjects were enrolled from the families of outpatients and medical staff and comprised the normal group, or the control group. Multi-slice computed tomography (CT) was performed at 1.0 mm thicknesses from the most proximal part of the pelvis to the point 2 cm inferior to the tibial tuberosity to reconstruct a 3D bone model from the pelvis to the proximal tibia for each subject using a helical scanner (Aquilion, Toshiba, Tokyo, Japan). CT was performed at 120 kVp and 150 mAs with the patient in the supine position and knees fully extended. For the DDH group, CT was performed before surgery for planning of curved periacetabular osteotomy. This study was approved by the institutional research board of our institution (No. 2016–0067), and written informed consent was obtained from the participants of the normal group. For the DDH group, informed consent was waived by the same institutional research board of our institution because this was a cross-sectional study (approval no. 2016–0067).

### Measurements

ZedHip® software (Lexi, Tokyo, Japan) was used to reconstruct accurate 3D bone models of the femur and tibia [14, 15]. The 3D model of the femur was adjusted to the plane that includes the most posterior point of the bilateral posterior condyles and the most posterior point of the greater trochanter, the so-called retrocondylar plane [16], as the coordinate system of the femur.

**Fig. 1:**
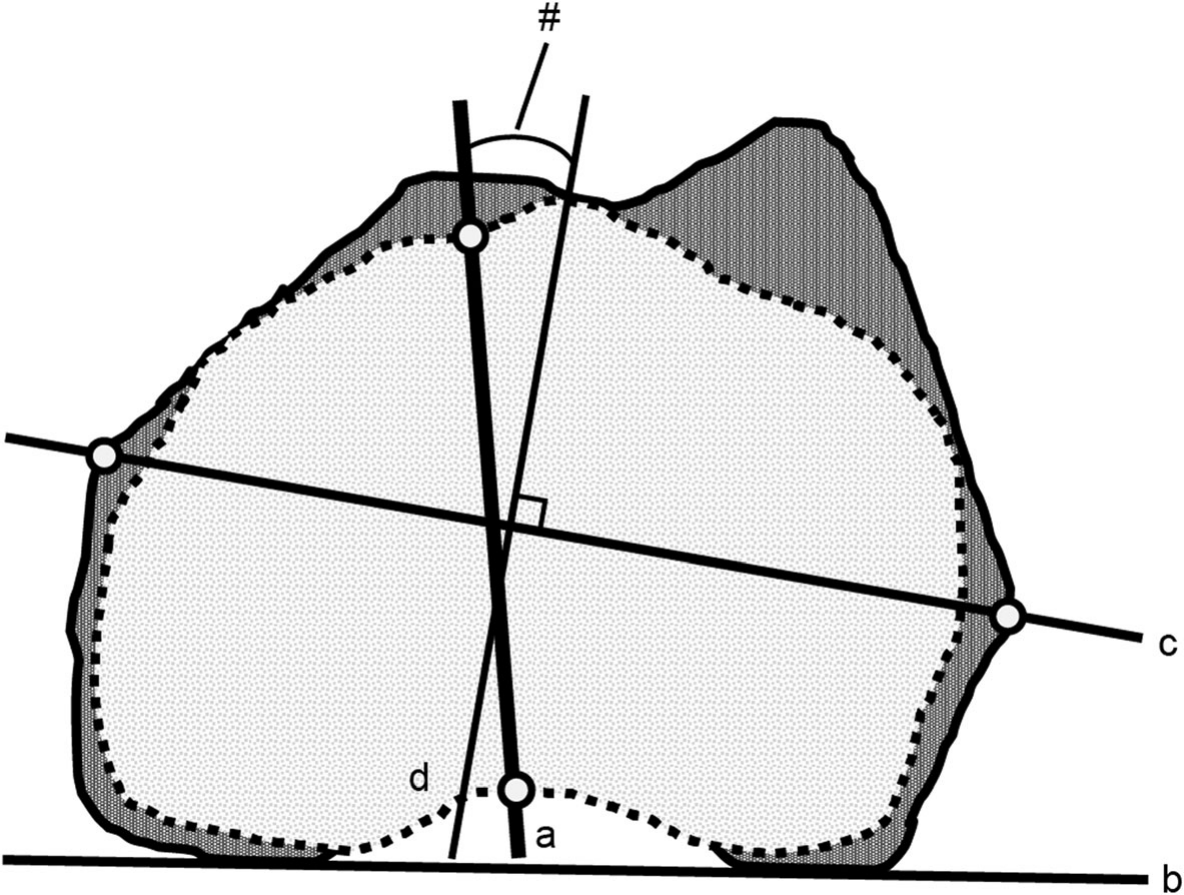
Measurement of the knee rotation angle. Knee rotation angle (#) is the angle between the tibial anteroposterior axis (**a**) and the line perpendicular to the clinical epicondylar axis (**d**). The solid line and dotted line represent the contour of the femoral condyle and tibial condyle projected onto the femoral horizontal plane, respectively. Clinical epicondylar axis (**c**) and posterior condylar axis (**b**)

The femoral neck axis was measured according a previous report by Sugano in the plane just below the femoral head [17]. Femoral neck anteversion (FNA) was defined as the angle formed by the femoral neck axis as above and the posterior condylar axis (PCA). The clinical epicondylar axis (CEA) was defined as the line formed by the most prominent points of the medial and lateral epicondyles. CEA length was defined as the distance between the most prominent points of the medial and lateral epicondyles. The condylar twist angle (CTA) was defined as the angle formed by the CEA and PCA; a positive CEA value meant that the CEA was externally rotated relative to the PCA [18]. The measurements of the CEA and CTA were also performed in the coordinate system of the femur. The line through the midpoint of the most prominent points of the medial and lateral epicondylar prominences projected on the axial plane of the coordinate system of the femur and the line perpendicular to the CEA was defined as the anteroposterior (AP) axis of the femur. The AP axis of the tibia was defined as Akagi’s line [19]. The KRA was defined as the angle connecting the AP axis of the femur and the tibia, projected onto the axial plane of the femoral coordinate system as above (Fig. 1). With regard to the KRA, positive values were defined as external rotation of the tibia relative to the femur, and negative values were defined as internal rotation.

**Fig. 2:**
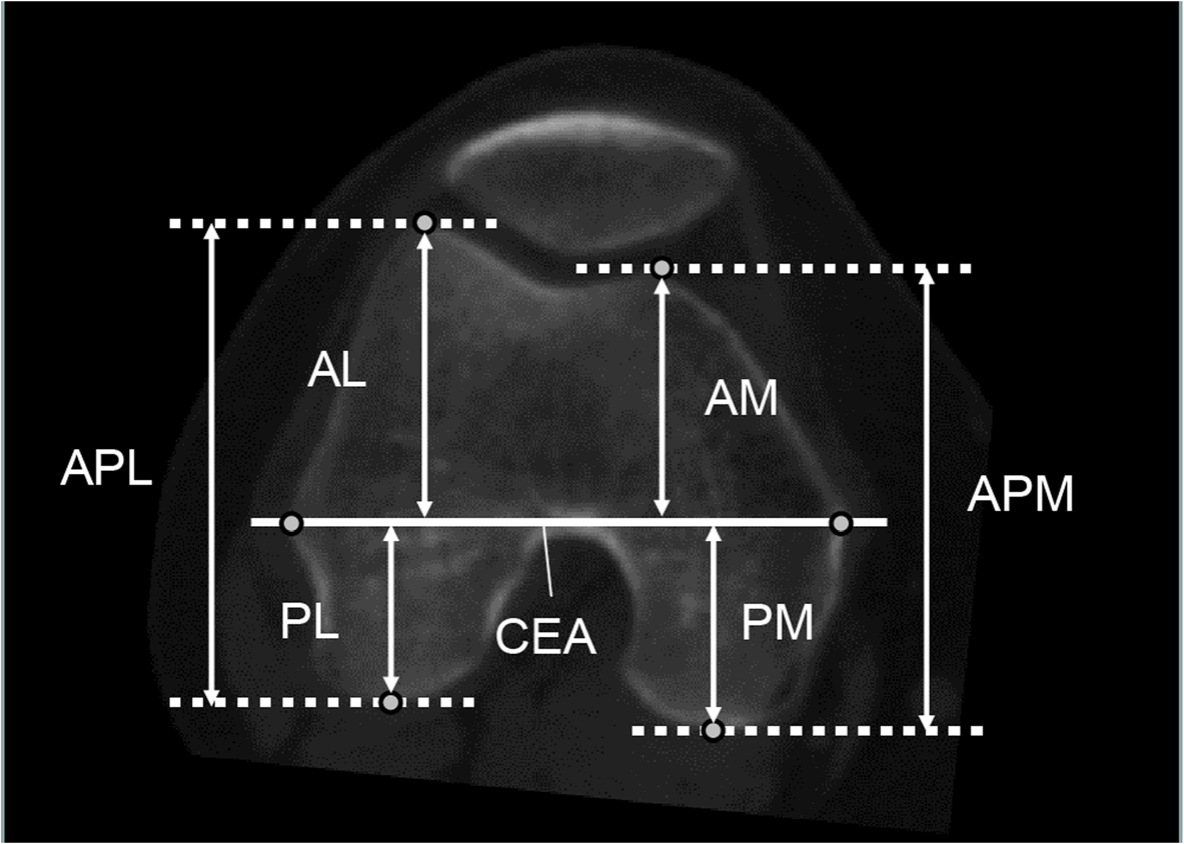
Measurement of the anteroposterior length of the femoral condyles. The anteroposterior dimensions of the clinical epicondylar axis (CEA) were measured. AM, AL: anterior part of the medial and lateral femoral condyles, respectively. PM, PL: posterior part of the medial and lateral femoral condyles, respectively. APM, APL: anteroposterior diameter of the medial and lateral femoral condyles, respectively. Therefore, the sum of the AM and PM was the APM, and the sum of the AL and PL was the APL

AP dimensions were also measured in both the medial and lateral condyles [10] (Fig. 2). A line was drawn at right angle to the CEA from the most anterior part of each anterior condyle. The anteroposterior dimension of the anterior part of the medial femoral condyle and that of the anterior part of the lateral femoral condyle were defined as AM and AL, respectively (Fig. 2). Similarly, the anteroposterior dimension of the posterior part of the medial femoral condyle and that of the posterior part of the lateral femoral condyle were defined as PM and PL, respectively (Fig. 2). Therefore, the sum of AM and PM (APM) was defined as the anteroposterior diameter of the medial femoral condyle, and the sum of AL and PL (APL) was defined as the anteroposterior diameter of lateral femoral condyle (Fig. 2). Condylar asymmetry was defined using the ratio between the APM and APL [20].

The parameters related to length, such as CEA length, AM, AL, PM, PL, APM, and APL, were standardized to body height by using the average body height of the normal group (154.4 cm): adjusted values = values of each parameter × (average body height of normal group (154.4)/body height of each person).

### Statistical analysis

We used SPSS statistical software (version 24, IBM Corp., Armonk, NY, USA) to analyze data. We used Pearson coefficients to determine the correlation coefficients of FNA, CTA, CEA length, KRA, AM, AL, PM, PL, APM, and APL. To assess the variation, we evaluated the mean absolute difference (MAD) and variability by standard deviation. We evaluated intraobserver reliabilities by intraclass correlation coefficients (ICCs) with two measurements by one observer at a minimum of 1-week interval and calculated a two-sided 95% confidence interval. Moreover, we assessed the interobserver reliability with a single measurement by two observers. We considered a *p*-value < 0.05 as statistically significant.

## Results

No statistically significant difference in body height, body weight, or BMI was found between the normal and DDH groups. The details of the research subjects are summarized in Table 1. FNA, KRA, and PL were statistically larger in the DDH group; by contrast, CEA length, AM, AL, and APM were significantly smaller (Table 1). APM was smaller in the DDH group, while APL was nearly equal in both groups, resulting in significantly less condylar asymmetry in the DDH group.

**Table 1:** Measurement of anatomical parameters in the normal and DDH groups

	Normal group (*n* = 90)	DDH group (*n* = 146)
FNA (deg)	18.0 ± 10.1***	28.2 ± 12.1***
CTA (deg)	7.1 ± 2.1	7.3 ± 1.8
KRA (deg)	1.6 ± 5.4***	9.0 ± 6.8***
CEA length (mm)	73.5 ± 3.3***	70.7 ± 2.9^a^***
AM (mm)	29.1 ± 2.1***	26.7 ± 2.0^a^***
PM (mm)	28.2 ± 1.6	27.7 ± 1.7^a^
AL (mm)	35.2 ± 2.4***	34.0 ± 1.8^a^***
PL (mm)	23.3 ± 1.8***	24.3 ± 1.7^a^***
APM (mm)	57.2 ± 2.9***	54.4 ± 2.9^a^***
APL (mm)	58.5 ± 2.5	58.3 ± 2.4^a^
Condylar asymmetry	0.98 ± 0.03***	0.93 ± 0.04***

*AL* anterolateral, *AM* anteromedial, *APL* anteroposterior length of lateral femoral condyle, *APM* anteroposterior length of medial femoral condyle, *CEA* clinical epicondylar axis, *CTA* condylar twist angle, *DDH* developmental dysplasia of the hip, *FNA* femoral neck anteversion, *KRA* knee rotation angle, *PL* posterolateral, *PM* posteromedial

Values are mean ± standard deviation.

*: *p* < 0.05, **: *p* < 0.01, ***: *p* < 0.001

^a^standardized by using the average body height of the normal group (154.4 cm)

**Fig. 3:**
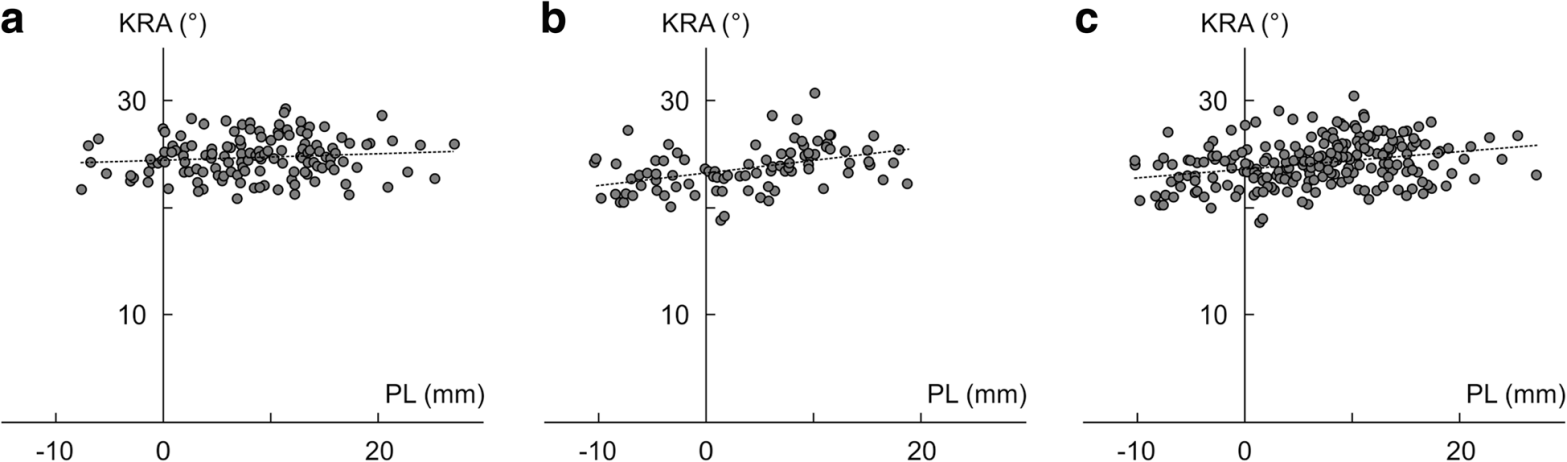
Correlation between the knee rotation angle and the length of the posterior part of the medial and lateral femoral condyles. With regard to the correlation between the knee rotation angle (KRA), the length of the posterior part of the medial and lateral femoral condyles (PL) was the highest in the normal group, DDH group, and total subjects

The correlation analysis demonstrated that CTA and PL length were significantly positively correlated with KRA in both the normal (CTA: *r* = 0.286, *p* = 0.007, PL: *r* = 0.429, *p* < 0.001) and DDH groups (CTA: *r* = 0.230, *p* = 0.033, PL: *r* = 0.272, *p* < 0.005) (Table 2) (Fig. 3a-c). Moreover, compared to other parameters, the correlation coefficient was the highest between KRA and PL in both the normal and DDH groups. In contrast, there was no statistical correlation between KRA and FNA in the DDH group (*r* = 0.094, *p* = 0.264), but there was a statistical correlation between KRA and FNA in the normal group (*r* = 0.243, *p* = 0.039) (Table 2). With regard to the validation, we obtained a high ICC for both intraobserver and interobserver reliabilities (Table 3).

**Table 2:** Correlation coefficient between KRA in the normal and DDH groups

	Normal group (*n* = 90)	DDH group (*n* = 146)	Total (*n* = 236)
FNA (deg)	0.243*	0.094	0.220*
CTA (deg)	0.286**	0.230*	0.250*
CEA length (mm)	−0.153	0.073	−0.187
AM (mm)	−0.089	−0.097	− 0.172
PM (mm)	−0.115	0.013	−0.080
AL (mm)	−0.230*	−0.128	− 0.237*
PL (mm)	0.429***	0.272**	0.358***
APM (mm)	−0.127	−0.062	− 0.114
APL (mm)	0.148	−0.114	−0.140

Values are correlation coefficient (*p* value).

*AL* anterolateral, *AM* anteromedial, *APL* anteroposterior length of lateral femoral condyle, *APM* anteroposterior length of medial femoral condyle, *CEA* clinical epicondylar axis, *CTA* condylar twist angle, *DDH* developmental dysplasia of the hip, *FNA* femoral neck anteversion, *KRA* knee rotation angle, *PL* posterolateral, *PM* posteromedial.

*: *p* < 0.05, **: *p* < 0.01, ***: *p* < 0.001

**Table 3:** Intra- and interobserver reliabilities of each parameter

	Intraobserver reliability		Interobserver reliability	
	MAD±SD	ICC	MAD±SD	ICC
FNA (deg)	1.28 ± 1.45	0.868	1.55 ± 1.82	0.822
CTA (deg)	0.64 ± 0.48	0.903	0.79 ± 0.57	0.874
CEA length (mm)	1.47 ± 1.72	0.841	1.87 ± 1.84	0.811
AM (mm)	1.57 ± 1.86	0.818	1.78 ± 1.93	0.806
PM (mm)	0.73 ± 0.52	0.873	0.86 ± 0.77	0.842
AL (mm)	1.24 ± 0.92	0.848	1.58 ± 1.34	0.827
PL (mm)	1.57 ± 1.86	0.818	1.78 ± 1.93	0.806
APM (mm)	0.73 ± 0.52	0.873	0.86 ± 0.77	0.841
APL (mm)	1.24 ± 0.92	0.838	1.58 ± 1.34	0.824

*AL* anterolateral, *AM* anteromedial, *APL* anteroposterior length of lateral femoral condyle, *APM* anteroposterior length of medial femoral condyle, *CEA* clinical epicondylar axis, *CTA* condylar twist angle, *DDH* developmental dysplasia of the hip, *FNA* femoral neck anteversion, *ICC* intraclass correlation coefficient, *KRA* knee rotation angle, *MAD* mean absolute difference, *PL* posterolateral, *PM* posteromedial, *SD* standard deviation

## Discussion

In this study, we found that subjects with a greater PL had a greater KRA. Thus, we speculated that in individuals with a greater PL, the contact point between the lateral femoral condyle and the lateral tibia plateau moved posteriorly; consequently, the tibia may be more externally rotated relative to the femur, possibly leading to a greater KRA.

A similar trend was observed between KRA and CTA in both the normal and DDH groups, but its influence was less than that of the PL length. In contrast, the DDH group had a greater FNA as well as a greater KRA, but these values may not have been morphologically associated.

There have been few investigations that focused on the characteristics of the knee joint in DDH patients [19]. Li et al. reported on the morphological features of the knee joints of DDH patients, including impaired development of femoral condyles. There was asymmetry of the medial and lateral condyles in DDH patients, and this may have led to a lateral shift of the patella and increased patellar tilt angle. Kandemir et al. [21] described significant valgus deformities of the knee in severe DDH patients compared to that in a control group. It appears that the pathological condition of the hip joint in DDH patients may cause developmental changes in the bony anatomy of the knee joint, such as a valgus deformity [21]. Several studies have described that femoral anteversion in the DDH group was greater (10–14°) than that of age-matched subjects [3], which are in agreement with the results of the present study. The CEA length and AP diameter of the medial condyle were decreased in the DDH group, while the AP diameter of the lateral condyle was similar to that of the normal group. Subsequently, the ratio of the AP diameter of the medial condyle to that of the lateral condyle decreased. These results were similar to those of Li et al. [6]. These findings of condylar dysplasia may be considered the morphological characteristics of DDH patients.

Morphological deformities in the femoral condyle may affect the location and the tilt of the patella [22]. Li et al. [6] reported that the lateral shift of the patella was smaller, and the patellar tilt angle was greater in DDH patients. Moreover, they described that compared to patients without DDH, the amount of bone cut from the anterior part of the lateral femoral condyle was likely greater than that of the anterior part of the medial condyle in total knee arthroplasty (TKA) [22] because of the greater anterior femoral angle; subsequently, they suggested 3° of lateral rotation of the femoral condyle for TKA in DDH patients [23]. The patellar instability was likely caused by the increased external rotation of the tibia. The external rotation of the tibia will obviously increase the side stress of the patella, which may cause anterior knee pain [6]. As written above, patients with larger KRA and PL will show a rotational mismatch after TKA, which might lead to postoperative knee pain, dissatisfaction, or an unstable feeling. Therefore, we considered that the external rotation of the femoral component and/or internal rotation of the tibial component can be a possible solution to this problem during TKA.

The current study has some strengths. First, in addition to evaluating DDH patients, we included 45 normal, healthy women as a control group, although they were somewhat older than those in the DDH group. Second, we used ZedHip® to examine the 3D digital bone models. The measurement of 3D digital bone models by using this software can provide accuracy and duplicability of the spatial relationship between the femur and the tibia within 1° and 1 mm of accuracy [15]. Therefore, we estimated that they were small projection errors and misalignment in the current study.

In contrast, this study has several limitations. First, the KRA was examined in the supine position. However, previous studies did determine that the difference in KRA between the supine and standing positions was negligible [14, 24]. Second, in this study, the scans were measured between the iliac crest and 2 cm under the tibial tuberosity; they did not include the whole length of the tibia. Therefore, we could not examine some parameters such as tibial varus or below in this study. Third, the strength of the correlation coefficient is moderate or weak. Fourth, there was a significant difference in age between the normal and DDH groups. It is unclear whether this difference affected the findings of this study; however, this is a known limitation. Lastly, we investigated only women in this study because DDH is more prevalent in women (women: men, 9:1) [25]. In our institution, fewer than 20 men with DDH underwent any surgery for over a period of 8 years. Several studies described no marked differences in femoral condyle geometry between men and women [26]. Therefore, we believe these results may apply also to men. However, a similar evaluation should be conducted in male subjects in the future.

## Conclusion

The morphology of the knee joint was affected by hip dysplasia. We found that the PL length was significantly positively correlated with KRA in both the normal and DDH groups, and this finding indicates that a greater PL leads to greater KRA. These findings of the existence of morphological changes in the knee joint may prove helpful when determining the alignment of implants for TKA in DDH patients.

## Data Availability

The datasets used and/or analyzed during the current study are available from the corresponding author on reasonable request.

## Abbreviations

AL: Anterolateral
AM: Anteromedial
APL: Anteroposterior length of lateral femoral condyle
APM: Anteroposterior length of medial femoral condyle
CEA: Clinical Epicondylar Axis
CTA: Condylar Twist Angle
DDH: Developmental Dysplasia of the Hip
FNA: Femoral Neck Anteversion
ICC: Intraclass Correlation Coefficients
KRA: Knee Rotation Angle
PCA: Posterior Condylar Axis
PL: Posterolateral
PM: Posteromedial
